# Clinical Characteristics of Infections Caused by *Streptococcus Anginosus Group*

**DOI:** 10.1038/s41598-020-65977-z

**Published:** 2020-06-03

**Authors:** Shenghua Jiang, Min Li, Tian Fu, Fenglian Shan, Luning Jiang, Zewei Shao

**Affiliations:** 1 Department of Respiratory and Critical Care Medicine, Affilitated Hospital of Jining Medical University, Jining, Shandong 272000 China; 2 Department of Respiratory and Critical Care Medicine, Jining NO.1 People’s Hospital, Jining, Shandong 272000 China; 3 Institute of Forensic Medicine and Laboratory Medicine, Jining Medical University, Jining, Shandong 272000 China

**Keywords:** Bacterial infection, Risk factors

## Abstract

This study aimed to investigate the clinical characteristics, distribution of different strains and risk factors of patients infected with *Streptococcus anginosus* group (SAG). In the population of 463 patients, the male-to-female ratio was 1.95:1, and the patient age ranged from 6 months to 103 years. There were 49 children (10.58%), 311 young and middle-aged adults (67.17%), and 103 elderly adults (22.25%). Approximately 45.4% had underlying conditions, which were mostly malignant tumors and diabetes. Of the 463 specimens, 254 were *S. anginosus* (54.86%), 173 were *S. constellatus* (37.37%), and 36 were *S. intermedius* (7.77%). According to the age distribution, the incidence peaked in the 35–54 year age group. Different sites of infection had statistically significant differences regarding the constituent ratios of these three species. Different age groups also exhibited statistically significant differences in constituent ratios of the pathogenic organisms, as well as organ infections. In our population, 269 were clinically cured, 184 reported satisfactory improvement, and 10 died. SAG, as an opportunistic pathogen, can induce pyogenic infections in patients of all ages and shows no significant gender predilection in any age group. The three pathogenic organisms had differences with respect to patient age and infections of body sites.

## Introduction

*Streptococcus anginosus* group (SAG) is a group of gram-positive streptococci normally colonizing the upper respiratory, digestive and reproductive tracts and consists of three distinct species, *S. anginosus*, *S. constellatus* and *S. intermedius*. SAG is not recognized as a causative pathogen. However, with the presence of certain incentive factors, colonized SAG directly induces noninvasive infections and causes invasive infections after entering normal sterile sites in the body, including the blood and serosal cavity, which eventually affect the tissues and organs of various systems of the body. The number of patients infected with SAG is increasing^1^. In fact, there is a lack of knowledge of these bacteria as opportunistic pathogens that may cause invasive infections^2^. Infections caused by SAG should be considered in the clinical diagnosis and treatment of related infections. On this basis, a retrospective analysis of patients diagnosed with infections with SAG admitted by the general teaching hospitals of two regional medical centers between Jan. 2014 and Nov. 2019 was conducted, and the findings are reported below.

## Materials and methods

### Clinical data

Patients with cultures positive for SAG (including *S. anginosus*, *S. constellatus* and *S. intermedius*) were collected from the bacterial laboratory database of the Affiliated Hospital of Jining Medical University Hospital and Jining No. 1 People’s Hospital, and blood, cerebrospinal fluid (CSF), bronchoalveolar lavage fluid, seroperitoneum, specimens by surgical drainage, mediastinum or soft tissue aspirate specimens were collected from these patients. A total of 463 subjects, including 306 males and 157 females, were enrolled in the present study after the clinical history of the patients was reviewed.

### Microbiological characteristics and bacterial identification

SAG consists of gram-positive, catalase-negative cocci and nonmotile facultative anaerobes that have typically small colonies (≤0.5 mm diameter) and demonstrate variable hemolysis patterns (alpha, beta, or gamma). *S. constellatus* is generally beta-hemolytic, while *S. intermedius* is mostly alpha-hemolytic. The presence of group F antigen in a small-colony-forming streptococcus isolated from a human specimen is likely a member of the SAG organisms. Bacterial identification: Samples were inoculated on blood agar, China-blue lactose agar, and chocolate agar (containing vancomycin) plates and cultured at 35 °C for 24 h until tiny pinpoint, circular, convex, alpha- or beta-hemolytic colonies with entire edges appeared. According to the Gram stain procedure, these bacteria were gram-positive cocci that were nonmotile, non-spore-forming, catalase-negative and arranged in pairs and in chains of varying lengths.

The identification of this bacterium in our laboratory is implemented by GP card (gram-positive bacteria identification card) of VITEK2 Compact, a fully automatic bacterial identification susceptibility instrument, and the flight mass spectrometer. Specific biochemical identification: the physiological and biochemical characteristics of SAG showed that urinase and sorbitol were negative; acetoin (VP) and arginine were positive. The species identification of SAG could be identified by the biochemical examination items including β-D fucosidase,β-D acetyl semi-glycosidase, neuraminidase and hyaluronidase. See the following table for details (Table 1).

**Table 1:** Key tests for species identification of streptococcus anginosus.

Name	β-D fucosidase	β-D acetyl semi-glycosidase	Neuramidinase	Hyaluronidase
*S. anginosus*	−	−	−	−
*S.constella* subsp constellatus	−	−	−	+
*S. constellatus* subsp pharynges	+	+	−	V
*S. intermedius*	+	+	+	+

“+“ indicates that more than 90% of the strains are positive, “−“ indicates that more than 90% of the strains are negative, and “V” indicates that 11% −89% of the strains are positive.

### Observations

Medical records of these patients were obtained from the medical record department, and the following data were collected: (1) general information such as sex and age, etc.; (2) basic conditions at presentation, including underlying diseases (such as multiple injuries, heart failure, central nervous system diseases, chronic respiratory disease, viral hepatitis, chronic renal insufficiency, tumors, blood disorders and diabetes), use of glucocorticoids and immunosuppressants 3 months before admission, and the presence of implants and surgical history in the last year. (3) laboratory test results of patients, including routine blood tests, C-reactive protein (CRP), serum albumin level (Alb) and bacterial culture; (4) type, dosage and date of antibiotics used; (5) infections, including the site, treatment and the presence of other bacterial infections; and (6) prognosis, i.e., the outcome (death or discharge after improvement) of the patients.

### Statistical analysis

Data were analyzed using SPSS 22 statistical software. Measurement data that fit a normal distribution are expressed by “x ± s”, while enumeration data are represented by the number of cases and constituent ratio. Univariate analysis was performed using the Q test (for measurement data). Chi-squared tests were performed to compare multiple constituent ratios; p ≤ 0.05 was considered statistically significant.

### Ethics statement

This study was approved by the Human Research Ethics Committee of the Affiliated Hospital of Jining Medical University. All patients were approached in accordance with approved ethical guidelines. They agreed to participate in this study and signed informed consent forms. We state that all methods in the study were performed in accordance with the relevant guidelines and regulations developed by the aforementioned ethics committee.

## Results

### Underlying diseases

210 of the 463 patients had major underlying diseases. Among them, 63 (30%) with solid tumors, 6 (2.86%) with hematological malignancies, 70 (33.33%) with type 2 diabetes mellitus (T_2_DM), 23 (10.95%) with central nervous system diseases (cerebral infarction, cerebral hemorrhage, brain trauma, myasthenia gravis, or Parkinson’s disease), 20 (9.52%) with chronic kidney failure, 12 (5.71%)with chronic respiratory disease, 6 (2.86%) with viral hepatitis, and 10 (4.77%)with connective tissue disease with oral hormone and immunosuppressive agents were found.

### Clinical manifestation

Most patients had fevers lasting for 1 to 33 days, including those with septicemia. In severe cases, ardent fever, chills, and systemic toxemia could be observed. Infections of the oropharynx were associated with odynophagia, cervicodynia, and trachelophyma, leading to difficulties in swallowing and opening the mouth. Large abscesses possibly resulted in upper airway obstructions. In the case of abscess-forming infection, there was a feeling of fluctuation during abscess formation. Patients with underlying conditions such as pneumonia and pulmonary abscess were likely to have respiratory symptoms such as coughing, shortness of breath, and rales; comorbidities associated with pleural effusion and mediastinal abscess were manifested by chest distress, chest pain, and even respiratory failure. When appendicitis occurred, in most cases, the pain shifted from the original site of onset to the right lower quadrant of the abdomen and lasted for long periods. Intra-abdominal abscess and peritonitis possibly induced ardent fever, abdominal pain, vomiting, and signs of peritoneal irritation; perianal abscess mainly produced painful swelling at the anus, while some patients might have experienced abscess diabrosis and watery discharge. When infection occurred in the extremities and subcutaneous tissue, there may have been local swelling, abscess diabrosis, and nonhealing wounds. In the case of diabetic foot disease as a comorbidity, clinical manifestations included diabetic foot ulcer and gangrene.

### Pathogenic organisms and sites of infections

This study had 463 positive cultures, including 254 for *S. anginosus* (54.86%), 173 for *S. constellatus* (37.37%), and 36 for *S. intermedius* (7.77%). Clinical diagnoses suggested infections in different body sites, which could be categorized as follows: cranial infection (5), oral and maxillofacial infection (43), ear-nose-throat (ENT) and cervical infection (61), chest infection (78), abdominal infection (134), pelvic infection (13), perianal infection (48), extremity and trunk (skin and soft tissue) infection (55), and occult infection (26). The abdomen was the most common site of infection, followed by the chest, ENT and neck. There were statistically significant differences regarding the constituent ratios of the SAG organisms (i.e., *S. anginosus*, *S. constellatus*, and *S. intermedius*) in different body sites of infection. By comparing the constituent ratios, it was noted that *S. constellatus* was more likely to cause infection of the thorax than of other body sites; *S. anginosus* had a closer association with cranial infection than infections of other body sites; *S. intermedius* infection was mostly found in the brain (Table 2).

**Table 2:** Constituent ratios of the three pathogenic organisms in different body sites.

species sites	cranial	oral and maxillofacial	ENT and cervical	chest	abdominal	extremity and trunk (skin and soft tissue)	pelvic	perianal	occult	p-value
	n = 5	n = 43	n = 61	n = 78	n = 134	n = 55	n = 13	n = 48	n = 26	
*S. constellatus* (n = 173)	1 (20.00)	22 (51.16)	22 (36.07)	42 (53.85)	47 (35.07)	15 (27.27)	3 (23.08)	13 (27.08)	8 (30.77)	0.003
*S. anginosus* (n = 254)	2 (40.00)	19 (44.19)	31 (50.82)	35 (44.87)	76 (56.72)	35 (63.64)	7 (53.85)	33 (68.75)	16 (61.54)	
*S. intermedius* (n = 36)	2 (40.00)	2 (4.65)	8 (13.11)	1 (1.28)	11 (8.21)	5 (9.09)	3 (23.08)	2 (4.17)	2 (7.69)	

Comparison between the oral cavity and extremities in the proportion of S. constellatus: P = 0.021, so Oral VS extremity: P = 0.021, oral VS extremity: P = 0.021, oral VS perianal: P = 0.030, ENT VS chest: P = 0.041, chest VS abdominal: P = 0.009, chest VS extremity: P = 0.003, chest VS perianal: P = 0.005, chest VS occult: P = 0.045, oral VS perianal in the proportion of S. anginosus: P = 0.021, chest VS extremity: P = 0.036, chest VS perianal: P = 0.01, cranial VS oral in the proportion of S. intermedius: P = 0.049, cranial VS chest: P = 0.009, cranial VS perianal: P = 0.04, ENT VS chest: P = 0.01, chest VS pelvis: P = 0.009.

### Association between patient age and pathogenic organisms and organs of infection

In the 463 patients, the male-to-female ratio was 1.95:1, and the patient age ranged from 6 months to 103 years. This study involved 306 males who accounted for 61.22%, 67.20%, and 64.08% of three separate age groups (namely, children, young and middle-aged adults, and senior adults). According to the age structure, the three age groups were further divided into children (0–17 years old), young and middle-aged adults I (18–34 years old), II (35–54 years old), III (55–64 years old), and senior adults (≥65 years old). In terms of patient age and onset of infection, patients aged 35–54 were exposed to the highest risk of infection (30.24%), followed by those aged 55–64 (23.76%). Different age groups also exhibited statistically significant differences in constituent ratios of the pathogenic organisms. According to the constituent ratios, we found that the constituent ratios of the three pathogenic organisms were different in the various age groups. *S. constellatus* infection (41.43%) was more common in patients aged between 35 and 54 than in other age groups; *S. anginosus* infection (68.75%) had a higher prevalence in those between the ages of 18 and 34 years old. For patients at the age of 65 and above, *S. intermedius* infection (11.65%) was a greater threat (Table 3).

**Table 3:** Constituent ratios of the three pathogenic organisms in the different age groups.

species age	<18	18–34	35–54	55–64	≥65	p-value
	n = 49	n = 61	n = 140	n = 110	n = 103	
*S. constellatus* (n = 173)	21 (42.86)	14 (22.95)	58 (41.43)	34 (30.91)	46 (44.66)	0.033
*S. anginosus* (n = 254)	24 (48.98)	42 (68.85)	73 (52.14)	70 (63.64)	45 (43.69)	
*S. intermedius* (n = 36)	4 (8.16)	5 (8.20)	9 (6.43)	6 (5.45)	12 (11.65)	

Comparison between <18 yr and 18–34 yr in the proportion of *S. constellatus*: P = 0.039, such as 18–34 yr vs 35–54 yr in the proportion of *S. constellatus*: P = 0.016, 18–34 yr vs ≥65 yr: P = 0.007, 55–64 yr vs ≥65 yr: P = 0 .047, <18 yr vs 18–34 yr in the proportion of *S. anginosus*: P = 0.05, 18–34 yr vs 35–54 yr: P = 0 .031,18–34 yr vs ≥65 yr: P = 0.002, 55–64 yr vs ≥65 yr: P = 0.004.

In terms of organ involvement, most cases were associated with infection of the appendix (103, 22.25%), followed by pulmonary infection (76, 16.41%). Differences with respect to patient age and the constituent ratios of associated organs of infection showed statistical significance. From the constituent ratios, it was found that infection of the appendix (27.18%) mostly occurred in patients at the age of 18 and below. For the group between 18 and 34 years of age, a higher proportion of anal infection (37.21%) was observed in comparison to that in other age groups. Oral and maxillofacial infection (53.85%) was the most commonly seen condition in those aged between 35 and 54. The prevalence of pulmonary infection was higher in patients aged 55 and above (34.21%, 55.26%) than in other age groups (Table 4).

**Table 4:** Constituent ratios of the age groups with infection of different organs.

Age Position	<18 yr	18–34 yr	35–54 yr	55–64 yr	≥65 yr	p-value
	N = 37	N = 44	N = 100	N = 75	N = 70	
cranial	2 (5.4)	1 (2.3)	1 (1.0)	0 (0.0)	1 (1.4)	<0.001
tonsil	4 (10.8)	9 (20.5)	11 (11.0)	9 (12.0)	7 (10.0)	
oral	0 (0.0)	4 (9.1)	14 (14.0)	3 (4.0)	5 (7.1)	
lung	1 (2.7)	0 (0.0)	7 (7.0)	26 (34.7)	42 (60.0)	
appendix	28 (75.7)	10 (22.7)	32 (32.0)	26 (34.7)	7 (10.0)	
anal	2 (5.4)	16 (36.4)	21 (21.0)	4 (5.3)	0 (0.0)	
hand and foot	0 (0.0)	4 (9.1)	14 (14.0)	7 (9.3)	8 (11.4)	

Comparison between <18 yr and 35–54 yr in the proportion of oral cases: P = 0.012. 35–54 yr vs 55–64 yr in the proportion of oral cases: P = 0.037. <18 yr vs 55–64 yr in the proportion of lung cases: P = 0, so <18 yr vs ≥65 yr: P = 0, 18–34 yr vs 55–64 yr: P = 0, 18–34 yr vs ≥ 65 yr: P = 0, 35–54 yr vs 55–64 yr: P = 0, 35–54 yr vs ≥65 yr: P = 0, 55–64 yr vs ≥65 yr: P = 0.003. <18 yr vs 18–34 yr in the proportion of appendiceal cases: P = 0, <18 yr vs 35–54 yr: P = 0, <18 yr vs 55–64 yr: P = 0, <18 yr vs ≥65 yr: P = 0, 35–54 yr vs ≥65 yr: P = 0.001, 55–64 yr vs ≥ 65 yr: P = 0.001, <18 yr vs 18–34 yr in the proportion of anal cases: P = 0.001, <18 yr vs 35–54 yr in the proportion of anal cases: P = 0.038, 18–34 yr vs 55–64 yr: P = 0, 18–34 yr vs ≥65 yr: P = 0, 35–54 yr vs 55–64 yr: P = 0.004, 35–54 yr vs ≥65 yr: P = 0. <18 yr vs 35–54 yr in the proportion of hand cases: P = 0.012, <18 yr vs ≥65 yr: P = 0.049.

### Laboratory test findings

White blood cell (WBC) count, CRP level, and serum albumin level were measured. Elevated WBC count and CRP level were observed in most patients, and no significant differences were observed between the 3 groups. The serum albumin level dropped to varying degrees, and the albumin level was significantly different between the elderly group and the other groups (P < 0.01) (Table 5).

**Table 5:** The laboratory features of patients classified into 3 groups.

group	number	WBC	CRP mg/L	Alb g/L
<18	49	13.55 ± 4.72	55.17 ± 40.27	42.49 ± 4.79
18–64	311	12.94 ± 5.52	75.34 ± 58.65	35.88 ± 7.66
≥65	103	13.56 ± 5.22	88.94 ± 59.02	31.55 ± 6.56

### Treatment and outcome

Based on drug sensitivity test results, diagnosed patients were given anti-infection treatment and invasive treatment (including surgery, puncture, drainage, etc.), nutritional support, or appropriate treatment for underlying diseases. In our population, 269 were clinically cured, 184 reported satisfactory improvement, and 10 died.

## Discussion

The anginosus group streptococci were first described by Guthof in 1956 after being isolated from dental abscesses. Since then, there have been reports on varied types of abscesses and relatively small studies on this subject. The incidence rate has reportedly increased continuously in the past two decades^1^. However, relevant data are not available on China’s part. To date, only limited clinical studies have presented detailed analyses of SAG^4–7^. However, these studies have investigated SAG organisms in children and adults separately. We retrospectively studied the clinical data of patients of all age groups infected with this pathogen to analyze the clinical characteristics of diseases due to SAG.

From the patients aged 6 months to 103 years in the present study, it was found that SAG bacterial infections could occur in individuals despite their ages and show a clear male predominance. The male-to-female ratios in the different age groups had no significant differences, which is similar to the results reported by several previously published studies^4,6,7^. By comparing the numbers of patients in all age groups, it was noted that adult patients at the age of 35–54 accounted for the largest proportion of our study population, which agrees with a previously reported study^6^. There were statistically significant differences regarding the constituent ratios of the three pathogenic organisms in the age groups. Based on the constituent ratios, the three pathogenic organisms showed an unequal distribution in every age group. *S. constellatus* had a higher proportion in patients aged between 35 and 54 than in other age groups; similarly, *S. anginosus* had the highest proportion in the group that was 18-34 years old compared to other age groups. The proportion of *S. intermedius* in patients aged 65 and above was higher than that in the other age groups.

In this study, 93.30% of the 463 patients had community-acquired infections; 210 (45.36%) had serious underlying conditions, including chest surgery, malignancy, nervous system disease, drinking problems, and diabetes, which were believed to make these patients susceptible to SAG^8–11^. Malignancy and diabetes were predominant among the underlying conditions, which is similar to previous studies^4,10^. However, the similar proportions of malignancy and diabetes might relate to the distribution of the underlying conditions. Among the patients with diabetes, 11 had diabetic foot disease, and 7 had subcutaneous abscesses, subcutaneous wounds, and nonhealing decubiti. Of the 13 patients with malignant tumors in their heads and necks, 9 had postlaryngectomy stomas, 2 had tongue cancer resection, 1 had hypopharynx cancer resection, and 11 received radiotherapy. All these factors seem to be closely associated with immunodeficiency, direct damage to the local mucous membrane, and disruption of the microbial balance in the oral cavity following radiotherapy^12,13^. In addition, the infection cases in this study mostly occurred after 2016, which is probably associated with the use of hormonal agents and immunosuppressants, active interventions, sample harvesting, and improvements in bacterial cultivation.

SAG bacterial infections often induce pyogenic infections such as superficial or deep soft-tissue infections and involve multiple organs. In this study, common superficial soft-tissue infections included infections of wounds, operative incisions, and pressure sores; deep soft-tissue infections included oral and maxillofacial infections, cellulitis, and necrotizing fasciitis. Chest infections could have produced pulmonary abscesses, pneumonia, pleural effusions, pyopneumothorax, and pleural fistulas. The conditions of patients with infection of the appendix progressed rapidly to abscesses and perforation and possibly resulted in peritonitis; perianal abscesses largely led to complex conditions. In these cases, the abdomen and chest were mainly involved, and most infections were caused by *S. anginosus*, which agrees with previously published studies^14,15^. However, the constituent ratios of the pathogenic organisms in different body sites exhibited statistically significant differences. *S. constellatus* was more likely to produce chest infection; *S. anginosus* was closely associated with perianal abscess; and *S. intermedius* was a major cause of cranial infection. However, further evidence is needed to support the point of view that these species are strongly associated with infections of different body sites^3,6^. Further analysis of the infected organs revealed that most cases were associated with purulent appendicitis, followed by pulmonary infection. The constituent ratios of organ involvement also differed among the age groups. In the patients under 18, most cases involved appendicitis; for those between 18 and 34, anal abscess was the most common infection; and oral and maxillofacial infection was found in most patients aged 35 to 54. The prevalence of pulmonary infection was higher in patients aged 55 and above (55–64, ≥65) than in other age groups.

Laboratory test results showed that in these patients, the white blood cell count increased, the CRP level generally increased, and the serum albumin level dropped to varying degrees. Although such parameters have not been discussed in the preceding studies, they may provide us with a new direction from which to look closely at relevant cases. Comparing the white blood cell counts and the CRP levels in different age groups, there was no significant difference. However, senior adults had a lower level of albumin, and the difference showed statistical significance.

Although SAG is not the most common cause of appendicitis, perianal abscess, parapneumonic effusion, and tonsillar abscess, it may complicate a patient’s condition with abscesses and lead to interventions that include invasive procedures such as surgery, puncture, and drainage. The main treatment options for these 463 patients included anti-infection treatment, invasive procedures and symptomatic supportive care. A total of 121 patients were given combined anti-infection treatment, and 341 were administered anti-infection monotherapy (oral or intravenous). Most patients had a favorable prognosis. Ten patients died, with a total mortality of 2.16%.

This study has a few limitations. First, this was a retrospective study limited by its non-multicenter design and relatively small sample size, and thus, the results might not be generalizable to other populations. Second, despite the observation of clinical practices, this study adopted sampling criteria and body-site categories that differed from those in previous studies.

## Conclusion

As an opportunistic pathogen, SAG was found to produce pyogenic infections in patients of all ages and exhibited a clear male predominance in all age groups where there was no significant difference regarding the male-to-female ratio. The three pathogenic organisms showed differences with respect to patient age and infections of body sites. These patients required puncture drainage, surgical debridement and anti-infection treatment, and most patients had a favorable outcome.
